# Evaluation of the Methemoglobin-Haptoglobin Protein Complex for Detoxification of Cyanide, Azide, and Sulfide in *In Vitro* Binding Models

**DOI:** 10.1021/acs.chemrestox.6c00228

**Published:** 2026-07-13

**Authors:** Griffin J. Beyer, Mohd Asim Khan, Quintin O’Boyle, Tanmay Salvi, Ana Carolina S Barbeta, Kenechukwu H. Adimorah, Andre F. Palmer

**Affiliations:** † William G. Lowrie Department of Chemical and Biomolecular Engineering, 2647 The Ohio State University, Columbus, Ohio 43210, United States; ‡ Department of Biomedical Engineering, The Ohio State University, Columbus, Ohio 43210, United States

## Abstract

Cyanide, azide, and sulfide are potent anion inhibitors of mitochondrial enzymes, causing rapid disruption of cellular energy metabolism and potentially fatal outcomes. Current treatments for these toxic anions are limited, highlighting the need for broadly effective antidotes. Methemoglobin (methHb), the ferric form of hemoglobin (Hb), can bind these ligands with high affinity, acting as a mechanistic scavenger that prevents their interaction with mitochondrial targets. Binding methHb to haptoglobin (Hp), an endogenous Hb-binding plasma protein, offers a strategy to enhance methHb stability and potentially increase circulation time while reducing potential oxidative side effects. Hence, in this work, we examine the detoxification potential of both cell-free methHb and methHb–Hp protein complexes against these anions using *in vitro* binding assays. Analyses by size exclusion high-performance liquid chromatography (SEC-HPLC) and dynamic light scattering (DLS) indicate that methHb-Hp complex formation exhibits a similar increase in molecular size relative to the Hp solution, while circular dichroism spectroscopy confirms maintenance of protein secondary structure. Kinetic studies using UV–visible spectroscopy and stopped-flow techniques reveal rapid and efficient sequestration of cyanide, azide, and sulfide by both methHb and methHb–Hp. These results demonstrate that Hp association stabilizes methHb without compromising its multiligand binding capacity, supporting its promise as a flexible platform for developing broad-spectrum toxic anion antidotes.

## Graphical abstract

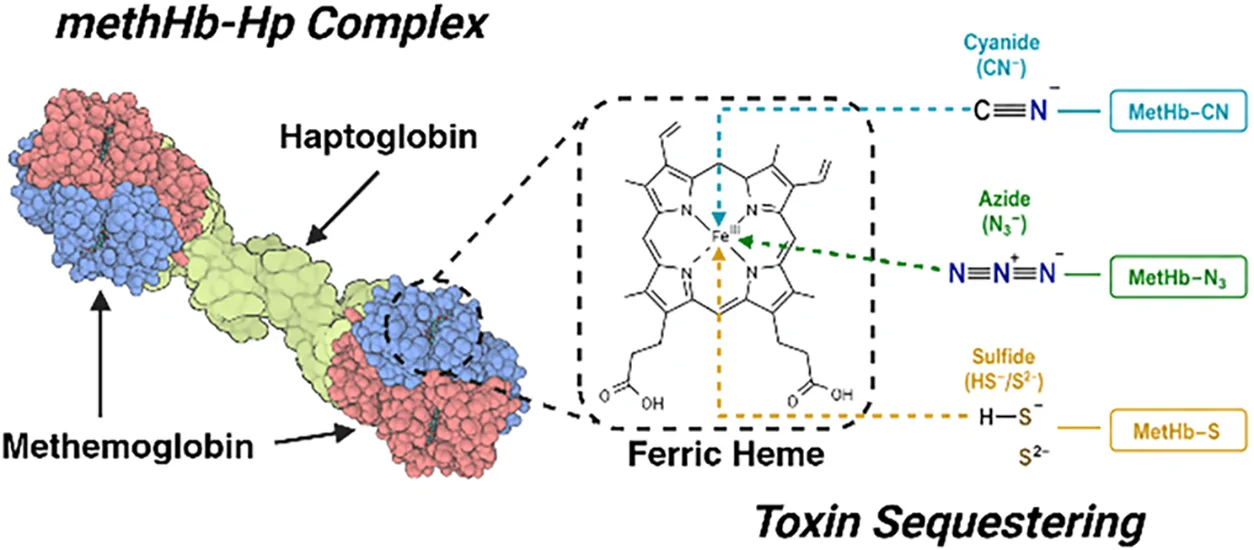

## 1 Introduction

Noncovalent stabilization of cell-free hemoglobin (Hb) or apohemoglobin (apoHb) in circulation, such as through complexing these molecules with the plasma glycoprotein haptoglobin (Hp), has emerged as a promising strategy to enhance the pharmacokinetics and safety of cell-free Hb and apoHb therapeutics.^1–6^ By forming Hb–Hp or apoHb–Hp protein complexes, Hb and apoHb are protected from rapid renal clearance, maintains the structural integrity of the bound Hb or apoHb, and preserves ligand-binding capacity, offering a versatile platform for neutralizing toxic small molecules.^3–6^ This approach is particularly attractive for addressing exposures to potent anionic toxins, including cyanide, azide, and sulfide, which pose significant industrial, environmental, and clinical hazards.^5,7–9^ These compounds are encountered across manufacturing, laboratory operations, mining, agriculture, and waste treatment, and even brief inhalation, ingestion, or dermal exposure can induce rapid and severe poisoning.^7–10^ Cyanide is widely employed in metal plating, plastic production, and fumigation,^9,11–13^ whereas hydrazoic acid and inorganic azides are used in propellants, detonators, and certain biosynthetic applications.^9,11–16^ Sulfide, generated in petrochemical processes, wastewater systems, and decaying organic matter, frequently accumulates as hydrogen sulfide gas in confined or enclosed spaces.^17,18^ Human exposures are often accidental, arising from fires, industrial accidents, sewage systems, or laboratory incidents,^7,8,12–14,17^ though intentional exposures, including suicide attempts, also contribute to their clinical relevance.

Despite their chemical diversity, cyanide, azide, and sulfide share a common mechanism of toxicity: inhibition of metalloproteins essential for mitochondrial respiration.^11–19^ Cyanide binds ferric iron (Fe^3+^) in cytochrome c oxidase, halting electron transport at complex IV.^11–13,19^ Azide similarly targets cytochrome c oxidase and interacts with catalase and other heme-containing enzymes, exacerbating mitochondrial dysfunction.^14–16^ Sulfide, in both protonated and unprotonated forms, impairs oxidative phosphorylation by forming strong complexes with heme centers in cytochrome c oxidase and reversibly inhibiting succinate dehydrogenase.^17,18^ These disruptions rapidly compromise cellular energy production, leading to lactic acidosis, seizures, cardiovascular collapse, and potentially death.^17,18,20^ The narrow therapeutic window and rapid symptom progression necessitate interventions capable of immediate detoxification of these anions.^17,18,20^

Current antidotes are limited and largely cyanide-specific. Hydroxocobalamin binds cyanide to form cyanocobalamin but is constrained by high plasma protein binding and ligand specificity.^21,22^ Nitrite-based therapies, often combined with thiosulfate, convert the Hb inside red blood cells (RBCs) to methemoglobin (methHb), which can sequester cyanide and promote its conversion to thiocyanate.^20,23–27^ However, these approaches reduce the oxygen-carrying capacity of RBCs and require careful monitoring to avoid excessive methemoglobinemia.^28,29^ Collectively, these approaches remain largely cyanide-specific, and while methHb can interact with other anions, nitrite-based therapies are not optimized to directly or efficiently neutralize azide or sulfide, leaving a significant gap in broad-spectrum antidotal coverage.^14,17,18,27^

Cell-free methHb, particularly when stabilized through methHb–Hp complex formation, represents a potentially promising broad-spectrum antidote due to the high affinity of its ferric heme iron for cyanide, azide, and sulfide.^20,23,25^ Previous studies exploring liposome-encapsulated methHb and methHb–albumin clusters have demonstrated effective detoxification of cyanide, azide, and sulfide *in vitro*, in cardiac cell models, and *in vivo* in mice.^30–35^ Importantly, Hp complexation also enables recognition by the macrophage scavenger receptor CD163, facilitating receptor-mediated endocytosis and clearance of the methHb–Hp complex bound to the toxic anion, which may mitigate off-target toxicity and support controlled systemic elimination.^4–6,36^ By leveraging this endogenous clearance pathway, methHb–Hp complexes may achieve prolonged circulation with improved safety while preserving multiligand binding capability. Therefore, in this work, we investigate methHb and methHb–Hp complexes as broad-spectrum scavengers for cyanide, azide, and sulfide. Through a combination of biochemical, biophysical, and kinetic analyses, we assess whether the methHb–Hp complex exhibits structural properties similar to ferrous Hb–Hp and preserves toxic anion detoxification potential, with the goal of establishing methHb–Hp as a next-generation platform for rapid neutralization of fast-acting mitochondrial toxins.

## 2 Materials and Methods

### 2.1 Materials

Sodium phosphate monobasic (NaH_2_PO_4_), sodium phosphate dibasic (Na_2_HPO_4_), sodium chloride (NaCl), potassium chloride (KCl), and sodium hydroxide (NaOH) were obtained from Fisher Scientific (Pittsburgh, PA). Potassium ferricyanide (K_3_Fe­(CN)_6_), potassium cyanide (KCN), sodium nitrite (NaNO_2_), and other reagents were also supplied by Fisher Scientific. Hollow fiber filter modules of varying sizes (0.65 μm, 500 kDa, 100, kDa, and 50 kDa) were provided by Repligen (Rancho Dominguez, CA). Expired human RBC units were obtained from Impact Life (Davenport, IA). Human Cohn fraction IV (FIV) was purchased from Seraplex (Pasadena, CA).

### 2.2 Hemoglobin Purification

Human Hb (hHb) was isolated from RBCs using tangential flow filtration (TFF) following established protocols.^37–40^ In brief, 20 units of packed human RBCs were diluted in 0.9 wt % saline to achieve a hematocrit of 20% ± 2%. The cell suspension was washed with 0.9 wt % saline across six diacycles using a 0.65 μm mPES hollow fiber filter. RBC lysis was then induced by adding an equal volume of 3.75 mM phosphate buffer (pH 7.4), and the mixture was stirred for 1 h. Cellular debris was removed by filtration through a 500 kDa hollow fiber module, and the resulting hemolysate was concentrated using a 50 kDa hollow fiber filter to a final hHb concentration of 200–250 mg/mL. Purified hHb was stored at −80 °C until further use.

### 2.3 Hemoglobin Oxidation and Purification

Oxidation of hHb was performed as described in the literature.^41,42^ Briefly, 10 g of hHb was diluted to 20 mg/mL and mixed in a 5:1 molar ratio of NaNO_2_ to heme. After 2 h, the reaction was stored at 4 °C overnight for purification the following day. The methHb was then purified via TFF using a 50 kDa hollow fiber filter for 8 diacylces with phosphate buffered saline (150 mM, pH 7.4) to remove excess NaNO_2_. The purified samples were concentrated to 50–100 mg/mL and stored at −80 °C until needed.

### 2.4 Haptoglobin Purification

Human Hp was isolated from Cohn Fraction IV (Seraplex, Pasadena, CA) using a TFF purification protocol described previously in the literature.^43–45^ Briefly. Cohn Fraction IV was homogenized via blender prior to lipid extraction with fumed silica. The solution was then centrifuged to remove the fumed silica and extracted lipids. The lipid free material was then subjected to a multimodule TFF train (0.2 μm, 100 kDa, and 50 kDa) to achieve purified Hp.

### 2.5 Hemoglobin–Haptoglobin Synthesis

hHb–Hp complexes were formed by mixing purified hHb and Hp in equimolar amounts based on the hHb binding capacity (HbBC) of the Hp, as previously described in the literature.^42,45–50^ Briefly, 1.5 g of hHb was mixed with Hp (HbBC = 36.3 mg/mL) such that hHb and Hp were incubated equimolar with respect to the HbBC of the Hp. For ferrous hHb, that corresponded to mixing 9 mL of a 161.8 mg/mL hHb solution with 41 mL of the Hp solution with an HbBC of 36.3 mg/mL. For ferric hHb, that corresponds to mixing 8 mL of a 177.8 mg/mL methHb solution with 41 mL of the Hp solution.

### 2.6 Protein Quantification (Cyanomethemoglobin Assay)

The concentrations of cell-free methHb and methHb–Hp were determined using UV–visible spectrophotometry, following established procedures.^37–40^ In brief, an HP 8452A diode array spectrophotometer (Olis, Athens, GA) was employed to quantify hemoglobin concentrations using the cyanomethemoglobin method.^51^

### 2.7 Molecular Weight and Size Quantification

Molecular weights were estimated using size exclusion chromatography (SEC) coupled to a high-performance liquid chromatography (HPLC) system, following previously reported protocols.^37–40^ Protein separation was carried out on an Ultimate 3000 HPLC equipped with an Acclaim SEC-1000 column (4.6 mm × 300 mm; Thermo Fisher Scientific, Waltham, MA). MethHb and methHb–Hp species were detected by monitoring the absorbance at 413 nm. In addition, hydrodynamic diameters were measured using dynamic light scattering (DLS) on a Malvern Zetasizer Pro (Malvern Panalytical Inc., Westborough, MA).

### 2.8 Circular Dichroism Spectroscopy

Circular dichroism (CD) measurements were performed using a JASCO J-815 spectropolarimeter (JASCO, Easton, MD). Far-UV spectra (190–260 nm), which assess protein secondary structure, were acquired at a concentration of 0.5 mg/mL (∼30 μM heme) in 150 mM PBS, pH 7.4, using a 0.1 cm quartz cuvette. Near-UV spectra (250–550 nm), reflecting protein tertiary structure, were recorded at 2.0 mg/mL (∼120 μM heme) under identical buffer and cuvette conditions. Each sample was scanned three times and the resulting spectra averaged. Baseline correction was performed by subtracting the buffer signal, and spectra are reported in millidegrees (mdeg).

### 2.9 Thermal Stability

Protein thermal stability was evaluated by monitoring the CD signal at 222 nm, which corresponds to α-helical content, over a temperature range of 25 to 90 °C. Samples were prepared at 0.5 mg/mL in 150 mM PBS (pH 7.4) and measured in a 0.1 cm quartz cuvette. The temperature was increased at 2.5 °C/min, with a 3 min equilibration at each set point before data acquisition. Baseline correction was performed using buffer-only measurements. Melting temperatures (*T*_m_) were determined by fitting the unfolding transitions to a sigmoidal model.

### 2.10 Haptoglobin Binding Kinetics

Hp binding kinetics were evaluated to investigate how structural modifications influence Hb recognition and potential clearance via the CD163 pathway, providing mechanistic insight into predicted *in vivo* behavior. Binding of Hp to hHb to form hHb–Hp or to methHb to form methHb–Hp was monitored through changes in intrinsic fluorescence emission at 310 nm (excitation 285 nm), which reflects formation of the Hp–Hb/Hp–methHb complex.^37–40^ Hp (0.25 μM) was incubated with varying concentrations of hHbor methHb (5, 10, 15, and 20 μM, tetramer basis). For each condition, ten kinetic traces were acquired and averaged. The resulting fluorescence time courses were fitted to a single-exponential model to extract pseudo–first-order rate constants. These constants were then plotted against the concentration of the hHb/methHb species, and the slope of the linear fit was used to calculate the second-order association rate constant (*k*_Hp–Hb_), expressed in μM^–1^ s^–1^.

Hb exchange of hHb–Hp and methHb–Hp with free hHb of the opposing redox state was also explored. The hHb–Hp and methHb–Hp complexes were mixed with equimolar free hHb (15 μM Hb basis) of the opposing redox state and incubated at room temperature for 1 h. Sample mixtures were analyzed using SEC-HPLC with inline UV–visible detection, allowing separation of the high-molecular-weight Hb–Hp complex from free Hb and enabling spectral extraction from each chromatographic peak. The Soret band of each SEC-HPLC peak was analyzed to confirm the redox state of each species.

### 2.11 Cyanide Binding Kinetics

Cyanide equilibrium binding experiments were conducted following previously reported protocols.^30–35,41^ Protein samples (hHb, hHb–Hp, methHb, and methHb–Hp) were prepared at a concentration of 8 μM on a heme basis. A 1 mM KCN stock solution was freshly prepared in 50 mM phosphate buffer (pH 7.4). Each protein sample was dispensed in 17 replicates of 250 μL per well in a 96-well plate. KCN was then added to achieve final concentrations of 0, 2, 4, 6, 8, 10, 12, 14, 16, 18, 20, 25, 30, 35, 40, 45, and 50 μM in each well. Plates were incubated at 25 °C for 1 h prior to measurement. Absorbance readings were taken at 405 nm (ferric heme) and 420 nm (cyanide-bound heme) using a plate reader. The absorbance at 405 nm was subsequently converted to fractional saturation (*Y*) according to eq 1.

1 $$Y=\frac{A_{0}-A}{A_{0}-A_{\infty }}$$

*A*_0_ represents the absorbance at 405 nm in the absence of KCN, *A* corresponds to the absorbance at a specific KCN concentration, and *A*_∞_ is the absorbance after the addition of excess ligand. Excess ligand was achieved by incubating each protein sample with 1 mM KCN. Saturation curves were generated by plotting fractional saturation (*Y*) against [KCN], and the apparent dissociation constant (*K*_d,app_) was determined by fitting the data to the Hill equation in the form shown in eq 2.

2 $$Y=\frac{{\left[L\right]}^{n}}{{K_{d}}^{n}+{\left[L\right]}^{n}}$$

In this model, [L] represents the ligand concentration, *n* reflects the cooperativity of binding, and *K*_d_ corresponds to the apparent dissociation constant. The *K*_d,app_ is equivalent to *S*_50_, the ligand concentration at which 50% of binding sites are occupied, providing a measure of the protein’s affinity for the ligand.

Rapid binding kinetics were measured using stopped-flow spectroscopy (SX-20, Applied Photophysics, Charlotte, NC) to determine the ligand association rate constant (*k*_on_, μM^–1^·s^–1^), following previously described procedures.^30–35,41^ MethHb and methHb–Hp were prepared at 5 μM on a heme basis, and KCN solutions were made at 50, 150, and 300 μM. Absorbance changes over time were monitored at 422 nm for each ligand concentration. Five kinetic traces were collected per replicate and averaged. The resulting time courses were fitted to a first-order rate model to extract pseudo–first-order rate constants for each KCN concentration. These pseudo–first-order constants were then plotted against KCN concentration, and a linear fit was used to calculate the second-order association rate constant (*k*_on_, μM^–1^·s^–1^). The dissociation rate constant (*k*_off_, s^–1^) was subsequently calculated as the product of *K*_d,app_ and *k*_on_.

### 2.12 Azide Binding Kinetics

Azide equilibrium binding experiments were performed following previously reported methods.^30–35,41^ Protein samples (hHb, hHb–Hp, methHb, and methHb–Hp) were prepared at 8 μM on a heme basis. A 1 mM NaN_3_ stock solution was prepared in 50 mM phosphate buffer (pH 7.4). Each protein sample was dispensed in 17 replicates of 250 μL per well in a 96-well plate. NaN_3_ was added to achieve final concentrations of 0, 2, 4, 6, 8, 10, 12, 14, 16, 18, 20, 25, 30, 35, 40, 45, and 50 μM per well. Plates were incubated at 25 °C for 1 h before measurement. The absorbance was recorded at 405 nm (ferric heme) and 418 nm (azide-bound heme), and the 405 nm values were converted to fractional saturation (*Y*) using eq 1. Saturation curves were generated by plotting *Y* versus [NaN_3_], and the apparent dissociation constant (*K*_d,app_) was determined by fitting the data to the Hill eq eq 2.

Rapid binding kinetics were assessed using stopped-flow spectroscopy (SX-20, Applied Photophysics, Charlotte, NC) to determine the association rate constant (*k*_on_, μM^–1^·s^–1^), following previously described protocols.^30–35,41^ MethHb and methHb–Hp were prepared at 5 μM heme, and NaN_3_ solutions were prepared at 50, 150, and 300 μM. Absorbance changes over time were monitored at 418 nm. Five kinetic traces were collected per replicate and averaged. The resulting curves were fitted to a first-order rate model to obtain pseudo–first-order rate constants at each ligand concentration. These constants were plotted against NaN_3_ concentration, and a linear fit was used to calculate the second-order association rate constant (*k*_on_, μM^–1^·s^–1^). The dissociation rate constant (*k*_off_, s^–1^) was calculated as the product of *K*_d,app_ and *k*_on_.

### 2.13 Sulfide Binding Kinetics

Sulfide equilibrium binding experiments were performed following previously reported protocols.^30–35,41^ Protein samples (hHb, hHb–Hp, methHb, and methHb–Hp) were prepared at 8 μM on a heme basis. A 1 mM Na_2_S stock solution was prepared in 50 mM phosphate buffer (pH 7.4). Each protein sample was dispensed in 17 replicates of 250 μL per well in a 96-well plate. Na_2_S was added to achieve final concentrations of 0, 2, 4, 6, 8, 10, 12, 14, 16, 18, 20, 25, 30, 35, 40, 45, and 50 μM per well. Plates were incubated at 25 °C for 1 h before measurement. The absorbance was recorded at 405 nm (ferric heme) and 426 nm (sulfide-bound heme), and the 405 nm values were converted to fractional saturation (*Y*) using eq 1. Saturation curves were generated by plotting *Y* versus [Na_2_S], and the apparent dissociation constant (*K*_d,app_) was determined by fitting the data to the Hill eq eq 2.

Rapid binding kinetics were assessed using stopped-flow spectroscopy (SX-20, Applied Photophysics, Charlotte, NC) to determine the association rate constant (*k*_on_, μM^–1^·s^–1^), as described previously.^30–35,41^ MethHb and methHb–Hp were prepared at 5 μM heme, and Na_2_S solutions were made at 50, 150, and 300 μM. Absorbance changes over time were monitored at 426 nm. Five kinetic traces were collected for each replicate and averaged. The averaged time courses were fitted to a first-order rate model to obtain pseudo–first-order rate constants for each sulfide concentration. These constants were plotted against Na_2_S concentration, and a linear fit was used to calculate the second-order association rate constant (*k*_on_, μM^–1^·s^–1^). The dissociation rate constant (*k*_off_, s^–1^) was calculated as the product of *K*_d,app_ and *k*_on_.

### 2.14 Statistical Analysis

All data are presented as the mean ± standard deviation, where applicable. Statistical comparisons were conducted using Student’s *t* test, one-way ANOVA, and Tukey’s post hoc test as appropriate, implemented in JMP software (JMP Student Pro, Version 18; SAS Institute Inc., Cary, NC). A significance threshold of 0.05 (α = 0.05) was applied for all analyses.

## 3 Results and Discussion

Table 1 summarizes the key biophysical and biochemical characteristics of hHb, hHb–Hp, methHb, and methHb–Hp. Included are molecular weight (MW) estimates from SEC-HPLC analysis, hydrodynamic diameters measured by DLS, Hp-binding kinetics, thermal melting temperatures (*T*_m_), and equilibrium binding parameters for cyanide, azide, and sulfide.

**Table 1:** Biophysical and Biochemical Parameters for MethHb, MethHb–Hp, and Their Precursors

Parameter		hHb (*n* = 3)^40^	hHb–Hp (*n* = 3)	methHb (*n* = 3)^41^	methHb–Hp (*n* = 3)
Concentration (mM)		3.23 ± 0.30	0.46 ± 0.01	1.30 ± 0.08	0.46 ± 0.01
metHb (%)		0.95 ± 0.45^b^ ^c^ ^d^	3.3 ± 0.3^a^ ^c^ ^d^	79.6 ± 9.1^a^ ^b^ ^d^	86.9 ± 6.5^a^ ^b^
*D*_eff_ (nm)		5.4 ± 0.1^b^ ^d^	15.4 ± 0.3^a^ ^c^	5.0 ± 0.5^b^ ^d^	15.8 ± 0.2^a^ ^c^
Average MW (kDa)		64.5 ± 0^b^ ^d^	650 ± 30^a^ ^c^	64.5 ± 0^b^ ^d^	700 ± 20^a^ ^c^
*k*_Hb–Hp_ (μM^–1^ s^–1^)		0.12 ± 0.01^b^ ^d^	0.0009 ± 0.00005^a^ ^c^	0.12 ± 0.01^b^ ^d^	0.001 ± 0.0001^a^ ^c^
*T*_m_ (°C)		64.2 ± 0.2^a^ ^b^ ^c^	85.5 ± 0.6^a^ ^c^	58.1 ± 0.2^a^ ^b^ ^d^	85.5 ± 0.7^a^ ^c^
Cyanide Binding	*K*_d,app_ (μM)	>50	>50	4.3 ± 0.6^d^	21.8 ± 2.8^c^
	*n*	N/A	N/A	1.9 ± 0.2^d^	1.3 ± 0.1^c^
	*k*_on_ (M^–1^ s^–1^)	≈0	N/A	82.7 ± 7.6^d^	134 ± 14^c^
	*k*_off_ (s^–1^)	N/A	N/A	3.5 × 10^–4^ ± 0.5 × 10^–5^ ^d^	2.9 × 10^–3^ ± 0.6 × 10^–3^ ^c^
Azide Binding	*K*_d,app_ (μM)	>50	>50	6.2 ± 0.6	7.8 ± 0.3
	*n*	N/A	N/A	1.4 ± 0.1^d^	1.7 ± 0.1^c^
	*k*_on_ (M^–1^ s^–1^)	≈0	N/A	58.3 ± 4.1^d^	98.8 ± 8.5^c^
	*k*_off_ (s^–1^)	N/A	N/A	3.6 × 10^–4^ ± 2.5 × 10^–5^ ^d^	7.7 × 10^–4^ ± 0.9 × 10^–4^ ^c^
Sulfide Binding	*K*_d,app_ (μM)	>50	>50	16.0 ± 0.9	19.4 ± 1.2
	*n*	N/A	N/A	1.9 ± 0.1^d^	1.5 ± 0.1^c^
	*k*_on_ (M^–1^ s^–1^)	≈0	N/A	9.4 ± 1.1^d^	63 ± 15^c^
	*k*_off_ (s^–1^)	N/A	N/A	1.5 × 10^–4^ ± 1.7 × 10^–5^ ^d^	1.2 × 10^–3^ ± 0.2 × 10^–3^ ^c^

a Significantly different with respect to hHb.

b hHb–Hp.

c methHb.

d methHb–Hp.

### 3.1 hHb–Hp and methHb–Hp Batch Parameters

hHb and methHb, both free and complexed with Hp, were prepared in consistent batches. Hp complexation of hHb and methHb increased molecular size and thermal stability, while methHb-containing samples retained high oxidation levels and measurable ligand-binding activity, in contrast to negligible binding by cell-free hHb. Overall, the batches were well-characterized and suitable for downstream biochemical analyses.

### 3.2 Molecular Weight and Size Quantification

The size/MW of hHb, hHb–Hp, methHb, and methHb–Hp were analyzed by SEC-HPLC to evaluate structural integrity and the effects of complexation and oxidation. All samples yielded well-resolved chromatograms on the Acclaim SEC-1000 column, with the absorbance monitored at 280 nm to detect all protein species as well as at 405/413 nm to selectively detect ferric/ferrous heme-containing species, respectively (Figure 1A,1B).

**Figure 1:**
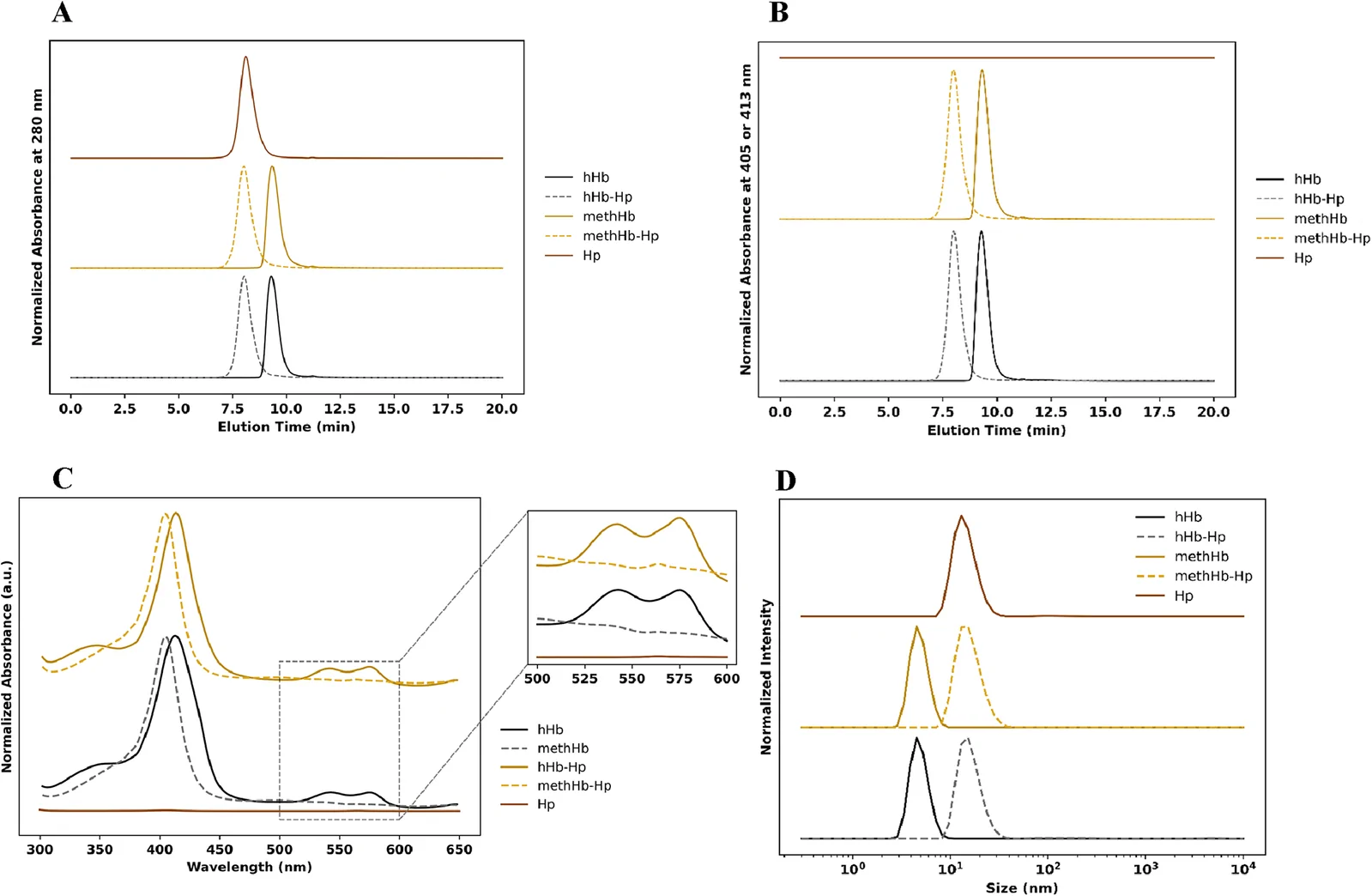
(A) Representative SEC-HPLC elution profiles of hHb, hHb–Hp, and their met derivatives at 280 nm for comprehensive protein analysis and (B) at 405 or 413 nm for heme containing species. (C) UV–visible spectra of hHb, hHb–Hp, and their met derivatives. (D) Representative dynamic light scattering (DLS) measurements showing hydrodynamic diameter distributions for the same species.

Consistent with expectations, native hHb eluted as a narrow peak corresponding to the tetrameric molecular weight (∼9.3 min, 64.5 kDa). In contrast, hHb–Hp samples exhibited a broader, earlier-eluting peak (∼8 min, 650 ± 30 kDa, *p* < 0.001), reflecting heterogeneous high–molecular-weight complexes formed through Hp binding. The earlier retention times relative to native hHb correspond to larger hydrodynamic radii, confirming successful formation of hHb–Hp complexes and virtually no unbound hHb.

Oxidation to the ferric state did not substantially change the chromatographic profiles of hHb–Hp. MethHb–Hp (700 ± 20 kDa, *p* > 0.05) retained a similar elution position and peak width compared to ferrous hHb–Hp with virtually no unbound methHb, indicating that conversion of Fe^2+^ to Fe^3+^ does not significantly perturb overall complex size. Native hHb also remained a single, well-defined peak after oxidation, demonstrating that formation of methHb does not promote aggregation or dimerization under these conditions.

UV–visible spectroscopy revealed a Soret band shift from ∼413 to 405 nm upon oxidation, consistent with conversion from ferrous to ferric heme (Figure 1C). The Q-band region also flattened in methHb–Hp compared to hHb–Hp, confirming effective oxidation.

Hydrodynamic diameters measured by DLS supported the SEC observations (Figure 1D). Native hHb exhibited a unimodal size distribution consistent with the tetrameric protein (5.4 ± 0.1 nm). hHb–Hp complexes displayed markedly larger average diameters (15.4 ± 0.3 nm) with broader polydispersity, in agreement with the SEC-HPLC data. Oxidized methHb–Hp showed no significant shift in particle size relative to its ferrous counterpart (15.8 ± 0.2 nm, *p* > 0.05), further confirming that oxidation does not disrupt complex architecture or promote additional association. The concordance between SEC-HPLC and DLS measurements indicates that hHb–Hp complexes are structurally stable and maintain consistent size characteristics under oxidative conditions.

Overall, these data indicate that Hp binding substantially increases the effective molecular size of hHb, and that these complexes remain structurally robust following oxidation to methHb. Such stability is essential for developing hHb–Hp or methHb–Hp–based therapeutics that must retain structural and functional integrity across physiological and oxidative conditions. The marked increase upon Hp complexation also carries important implications for vascular safety. While ferrous hHb is a highly efficient nitric oxide scavenger and potent vasoconstrictor, the larger hydrodynamic diameters of Hb–Hp and methHb–Hp is expected to restrict extravasation through the endothelial barrier, thereby limiting access to the vascular smooth muscle NO pool and reducing the risk of vasoconstriction.^52^ Although methHb itself retains some NO activity, its reduced intrinsic scavenging efficiency relative to ferrous hHb, combined with the steric constraints imposed by Hp complexation, represents a meaningful safety advantage for *in vivo* applications.

### 3.3 Circular Dichroism Spectroscopy

CD spectroscopy was used to assess the secondary and tertiary structures of hHb, hHb–Hp, and their met derivatives, shown in Figure 2.

**Figure 2:**
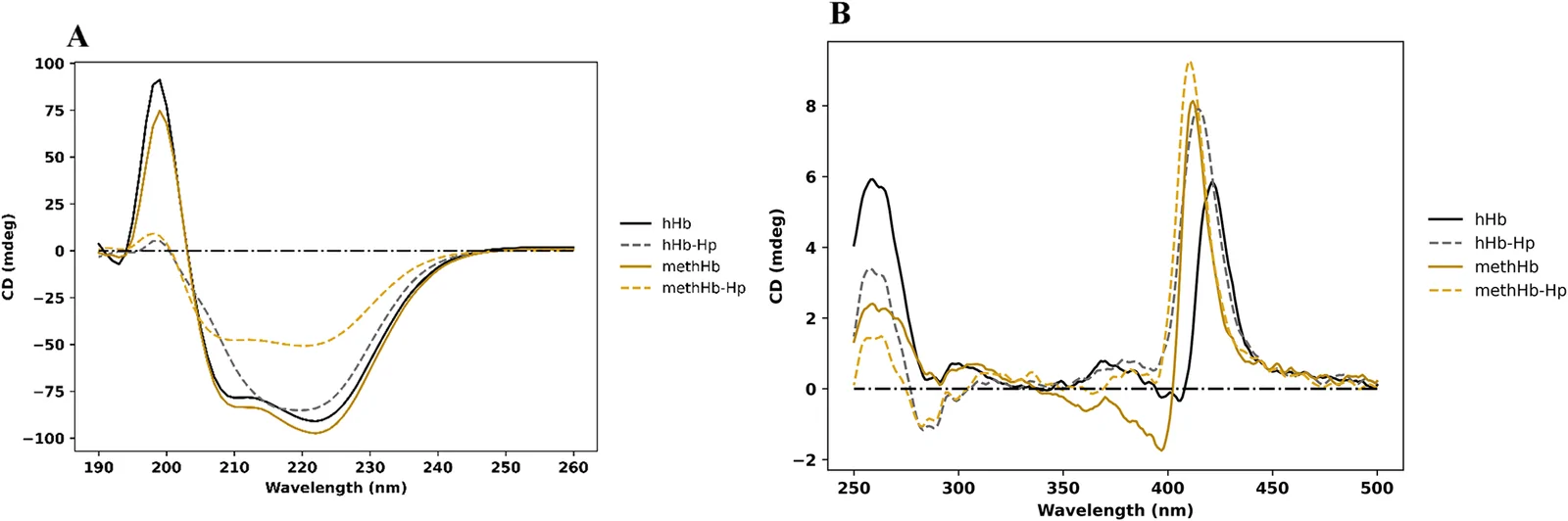
CD spectra of hHb, hHb–Hp, and their met derivatives. (A) Far-UV CD spectra (190–260 nm) assess protein secondary structure, primarily α-helical content, collected at 0.5 mg/mL (∼30 μM heme) in 150 mM PBS, pH 7.4. (B) Near-UV CD spectra (250–550 nm) assess protein tertiary structure at 2.0 mg/mL (∼120 μM heme) under identical buffer conditions. Spectra represent the average of three scans per sample after baseline correction against buffer controls and are reported as millidegrees (mdeg).

Far-UV circular dichroism spectra (Figure 2A) confirmed that all hHb samples are predominantly α-helical, with characteristic minima near 208 and 222 nm. Ferrous Hb–Hp complexes showed a convergence to a single minimum around 220 nm, suggesting a subtle reorganization of secondary structure upon Hp binding. In contrast, ferric Hb–Hp retained the overall spectral shape of uncomplexed hHb, with a modest decrease in signal intensity, indicating minimal perturbation of α-helical content upon oxidation. These observations demonstrate that Hp binding induces small adjustments in the Hb backbone in the ferrous state, while ferric hHb–Hp (i.e., methHb–Hp) preserves secondary structure compared to ferrous hHb–Hp.

Analysis of the near-UV CD region (Figure 2B), which reflects protein tertiary structure through signals from aromatic side chains and the heme group, revealed substantial overlap among all species. A modest shift in the 400–420 nm range was observed following oxidation to the ferric state, consistent with known changes in the heme Soret band. Aside from this expected spectral adjustment, the overall profiles remained largely unchanged, suggesting that the protein tertiary structure and heme pocket environment are preserved upon hHb–Hp complex formation and oxidation.

Taken together, these findings support that hHb maintains its structural integrity at both the secondary and tertiary levels after binding to Hp and undergoing oxidation. The small spectral change associated with ferric heme reflects normal electronic transitions rather than structural perturbation, indicating that methHb–Hp complexes remain structurally stable and suitable for further functional evaluation.

### 3.4 Thermal Stability

The thermal stability of hHb, hHb–Hp, and their met forms was evaluated by monitoring CD ellipticity at 222 nm as a function of temperature, providing a direct measure of α-helical unfolding, as shown in Figure 3.

**Figure 3:**
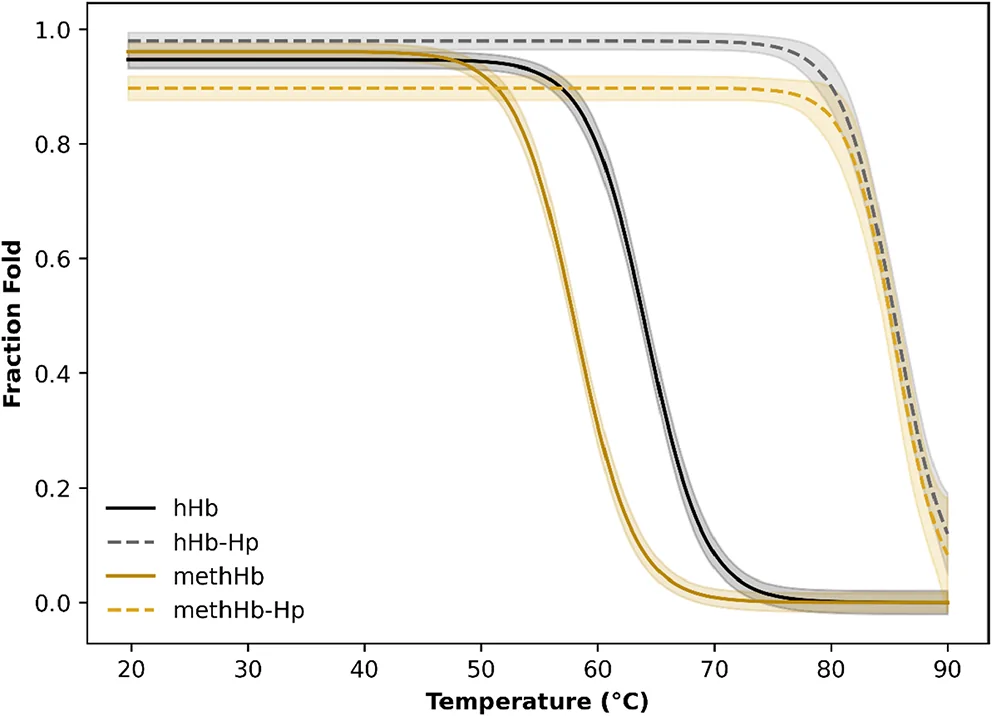
Thermal unfolding profiles of hHb, hHb–Hp, and their met derivatives measured by CD spectroscopy at 222 nm. Samples (0.5 mg/mL in 150 mM PBS, pH 7.4) were heated from 25–90 °C at 2.5 °C/min with a 3 min equilibration at each temperature. Sigmoidal fits to the unfolding transitions were used to determine melting temperatures (*T*_m_). *T*_m_ values are summarized in Table 1.

As previously reported, thermal denaturation analysis showed that native hHb undergoes a cooperative unfolding transition with a melting temperature (*T*_m_) of 64.2 ± 0.2 °C.^41^ Conversion to the ferric state significantly decreased protein stability, as metHb exhibited a lower *T*_m_ of 58.1 ± 0.2 °C (*p* < 0.0001).^41^ This reduction in thermal resilience is consistent with the destabilizing effects of ferric heme, which weakens subunit interactions and increases susceptibility to unfolding.

In contrast, formation of the hHb–Hp complex resulted in a pronounced increase in thermal stability. The hHb–Hp complex displayed a *T*_m_ of 85.5 ± 0.6 °C, representing a substantial enhancement relative to native hHb. This elevated stability suggests that binding to Hp provides significant structural reinforcement, likely through strong protein–protein interactions and shielding of vulnerable regions on the hHb surface. Notably, this stabilization effect exceeds that observed for unmodified hHb and indicates that complexation fundamentally alters the protein’s resistance to thermal perturbation.

Unlike native hHb, which is sensitive to oxidation, the hHb–Hp complex maintained its high thermal stability in the ferric state. MethHb–Hp exhibited a *T*_m_ of 85.5 ± 0.7 °C, showing no meaningful difference compared to the ferrous complex (*p* > 0.05). This result contrasts sharply with the behavior of unbound hHb and demonstrates that Hp binding effectively mitigates the destabilizing impact of heme oxidation. The preservation of thermal stability across redox states highlights the robustness of the Hb–Hp complex under conditions that would typically compromise protein integrity.

Overall, these results demonstrate that Hp association significantly enhances the thermal resilience of hHb while also protecting against oxidation-induced destabilization. The ability of the hHb–Hp complex to maintain structural integrity at elevated temperatures, regardless of heme redox state, underscores its potential utility in applications where protein stability is critical.

### 3.5 Haptoglobin Binding Kinetics

Hp–hHb binding kinetics were evaluated by monitoring intrinsic fluorescence quenching at 310 nm upon rapid mixing of Hp with increasing concentrations of hHb, hHb–Hp, methHb, or methHb–Hp. All reactions exhibited monoexponential fluorescence decay profiles, consistent with a single predominant binding event. Pseudo–first-order rate constants increased linearly with protein concentration, allowing calculation of the second-order association rate constant (*k*_Hp–Hb_) from the slope of the linear fits (Figure 4).

**Figure 4:**
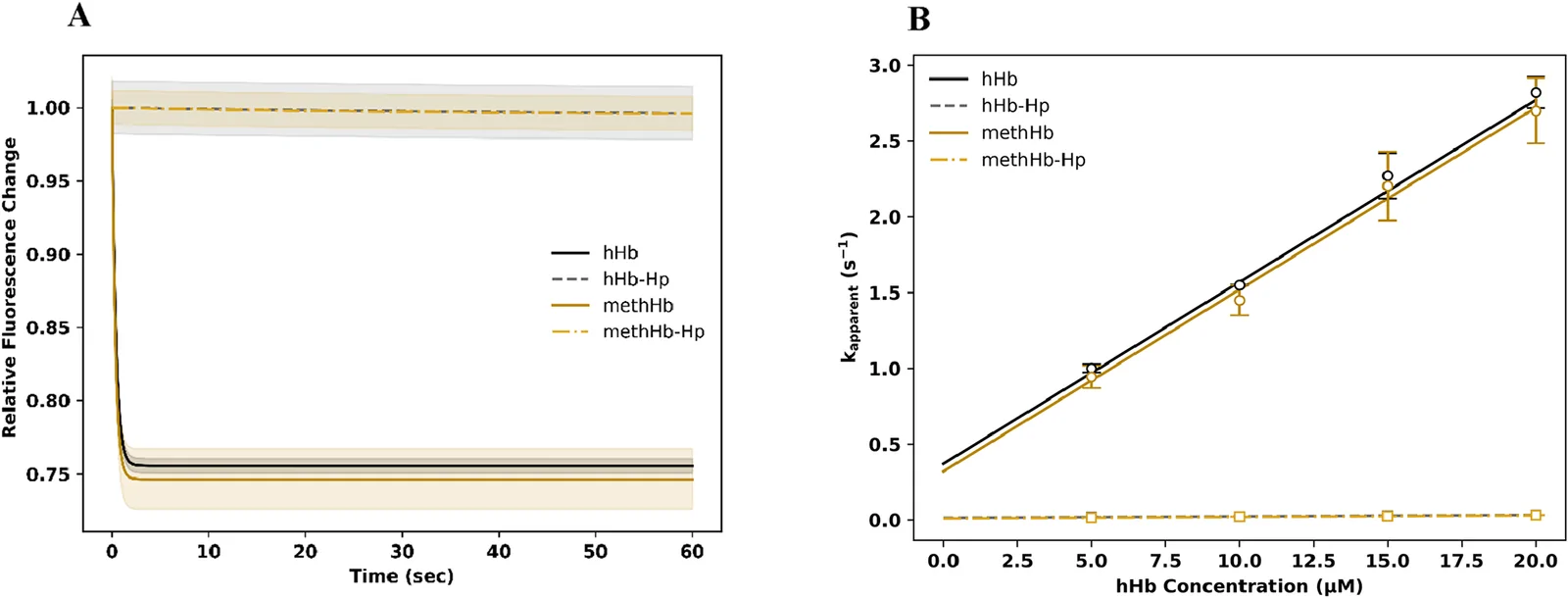
Association kinetics of Hp binding to hHb, hHb–Hp, and their met derivatives. Hp (0.25 μM) was mixed with increasing concentrations of hHb, hHb–Hp, or their corresponding met forms (5–20 μM, hHb tetramer basis). (A) Representative fluorescence decay at 310 nm (excitation 285 nm). Ten traces per condition were averaged prior to fitting. (B) Pseudo–first-order rate constants obtained from single-exponential fits were plotted against protein concentration to calculate second-order association rate constants (*k*_Hp–Hb_).

Previously reported data show that native hHb binds Hp with a rapid association rate constant of 0.12 ± 0.01 μM^–1^ s^–1^, and this value remains unchanged upon oxidation to methHb (0.12 ± 0.01 μM^–1^ s^–1^) (*p* > 0.05), indicating that the heme redox state does not influence Hp recognition or complex formation.^41^ This consistency suggests that the Hp-binding interface on hHb is largely unaffected by conversion from the ferrous to ferric state, preserving efficient interaction kinetics across both forms.

In contrast, the preformed hHb–Hp complex inherently reflects this high-affinity interaction and does not undergo further association with Hp in the same manner as cell-free hHb. Instead, the formation of the hHb–Hp complex represents a stabilized end point in which hHb is already sequestered by Hp. The reduced apparent association behavior observed for the hHb–Hp complex (0.0009–0.001 μM^–1^ s^–1^) likely reflects the absence of freely accessible binding interfaces, rather than a true decrease in intrinsic binding capability. This is consistent with the expectation that once hHb is bound within the Hp scaffold, the canonical Hb αβ dimer interface required for additional Hp engagement is no longer available. Importantly, oxidation of the hHb–Hp complex does not significantly alter this behavior. The methHb–Hp complex exhibits nearly identical kinetic values to its ferrous counterpart (*p* > 0.05), reinforcing that redox state does not play a major role in modulating further Hp interactions once the complex has formed. This parallels the behavior of cell-free hHb reported in the literature and further supports the conclusion that structural accessibility, rather than heme oxidation state, governs Hp binding dynamics.

The results of incubating ferrous Hb–Hp with free ferric hHb and incubating methHb-Hp with free ferrous hHb are shown in Figure 5. The size-exclusion chromatogram (Figure 5A) displays two distinct peaks corresponding to the hHb–Hp complex and free hHb. UV–visible spectra extracted from these chromatographic peaks (Figure 5B) revealed that the hHb–Hp fraction retains the spectral features characteristic of ferrous hHb, including a Soret maximum near ∼413 nm and visible α/β Q bands near ∼540 and ∼576 nm.^53^ In contrast, the free hHb peak exhibits spectral features consistent with ferric hHb, including a Soret maximum near ∼405 nm.

**Figure 5:**
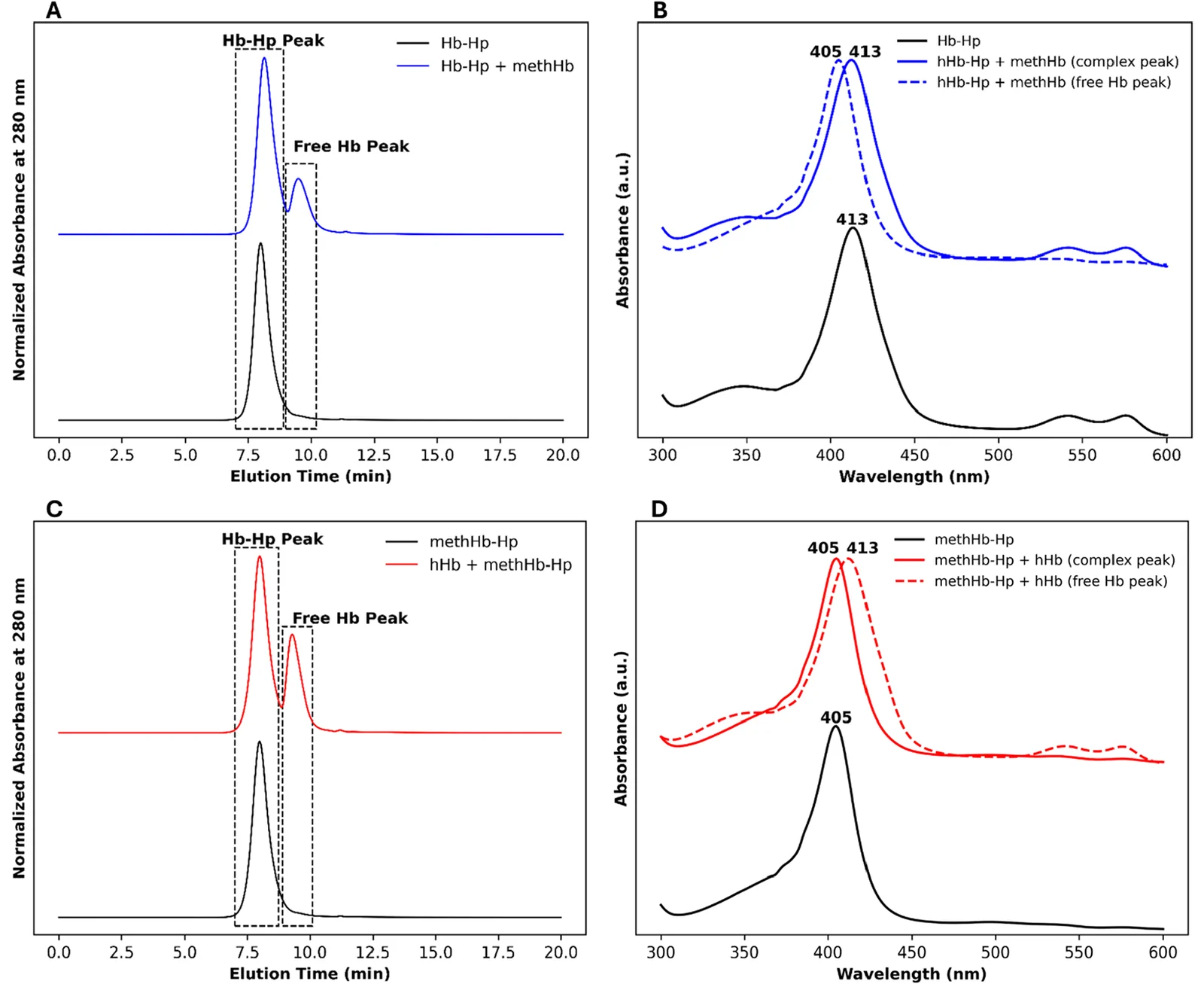
SEC-HPLC UV–visible analysis of ferrous/ferric Hb–Hp incubated with free ferric/ferrous Hb. (A) Representative size-exclusion chromatogram showing separation of the ferrous Hb–Hp complex and free ferric Hb. (B) UV–visible spectra extracted from the chromatographic peaks demonstrating that the Hb–Hp complex retains the ferrous Hb spectral signature while the free fraction corresponds to ferric Hb. (C) Representative size-exclusion chromatogram showing separation of the methHb–Hp complex and free ferrous Hb. (D) UV–visible spectra extracted from the chromatographic peaks demonstrating that the Hb–Hp complex retains the ferric Hb spectral signature while the free fraction corresponds to ferrous Hb.

The reciprocal experiment, in which ferric hHb–Hp complexes were incubated with free ferrous hHb, produced analogous results (Figure 5). The chromatogram (Figure 5C) again shows clear separation between the hHb–Hp complex and free hHb. Spectra extracted from the hHb–Hp peak (Figure 5D) display the characteristic ferric hHb Soret maximum near ∼405 nm,^53^ while the spectra associated with the free hHb peak exhibit the ferrous hHb signature with a Soret band near ∼413 nm and distinct visible α/β Q bands.^53^

Across both experimental conditions, hHb within the hHb–Hp complex retained its original oxidation state following incubation with free hHb of the opposite redox state. If hHb exchange were occurring, one would expect partial spectral conversion within the hHb–Hp peak due to incorporation of exchanged hHb αβ dimers from the free hHb pool. However, no spectral changes were observed. Additionally, no intermediate chromatographic peaks corresponding to partially exchanged hHb–Hp species were detected. The absence of such species further supports the conclusion that Hb bound within the hHb–Hp complex remains stable under the conditions examined.

These observations indicate that hHb bound to Hp does not undergo detectable exchange with free hHb in solution. This behavior is consistent with the exceptionally high affinity of the hHb–Hp interaction, which occurs in the subnanomolar range.^5,36^ Structural studies have shown that Hp binds hHb αβ dimers within a defined interface that stabilizes the complex and limits dissociation events.^5^ The stability of the hHb–Hp complex has important physiological implications. During hemolysis, Hp serves as a primary defense mechanism that rapidly captures extracellular Hb.^4,54,55^ The absence of hHb exchange suggests that once hHb is sequestered by Hp, it remains chemically isolated from the surrounding hHb pool in solution. Consequently, oxidized Hb captured by Hp is unlikely to equilibrate with ferrous hHb.

Taken together, these observations highlight a key distinction between cell-free hHb and Hp-bound hHb. While native hHb and metHb rapidly associate with Hp, the Hb–Hp complex represents a kinetically stable, prebound state with minimal capacity for further Hp interaction. This behavior underscores the effectiveness of Hp in sequestering hHb and limiting additional binding events, which has important implications for regulating hHb clearance and stability in biological systems. Further, hHb-based detoxification strategies, including engineered hHb therapeutics or ligand-scavenging systems, often rely on hHb in a defined oxidation state to selectively bind toxic small molecules such as cyanide, azide, or sulfide.^56,57^ Our observation that hHb–Hp complexes retain the original oxidation state of hHb suggests that once hHb is sequestered by Hp, its redox chemistry remains isolated from other extracellular hHb species in solution. This stability could help prevent unintended redox reactions that might compromise ligand-binding specificity or generate oxidative byproducts in therapeutic applications.

### 3.6 Cyanide Binding Kinetics

Cyanide binding kinetics were determined via equilibrium binding and rapid kinetic analysis via UV–visible spectroscopy and stopped-flow spectroscopy, respectively (shown in Figure 6).

**Figure 6:**
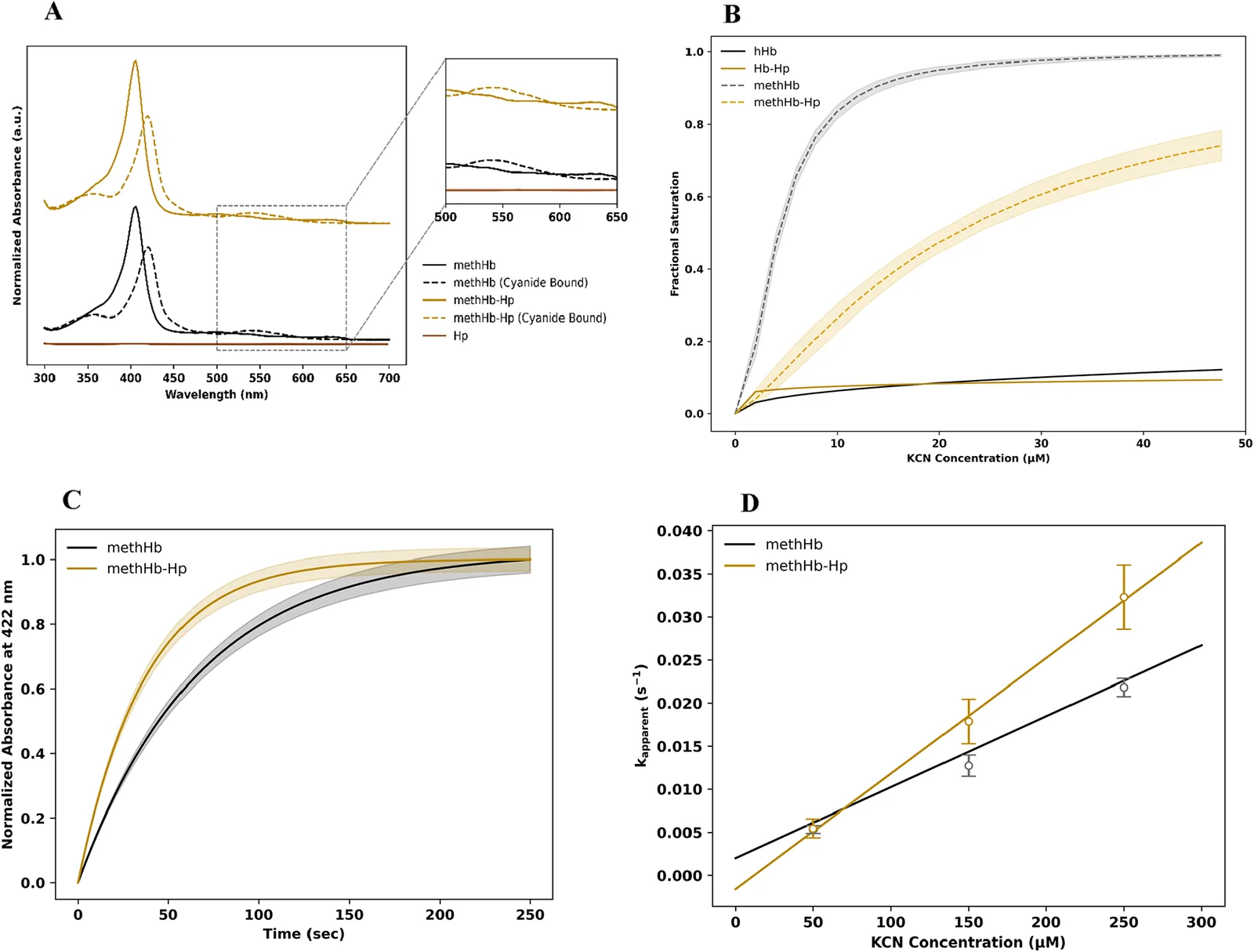
Equilibrium and kinetic analysis of cyanide binding to hHb-based constructs. (A) UV–visible spectra for methHb and methHb–Hp before and after cyanide binding. (B) Equilibrium cyanide binding curves for hHb, hHb–Hp, methHb, and methHb–Hp. Fractional saturation (*Y*) was calculated from changes in absorbance at 405 nm and plotted as a function of KCN concentration. Data were fit to the Hill equation to determine apparent dissociation constants (*K*_d,app_) and cooperativity coefficients where measurable. (C) Representative stopped-flow UV–visible spectroscopy kinetic traces for rapid binding of 100 μM cyanide with methHb and methHb–Hp. (D) Determination of cyanide association kinetics for methHb and methHb–Hp. Pseudo–first-order rate constants (*k*_app_), obtained from single-exponential fits of stopped-flow absorbance traces at 422 nm, were plotted against KCN concentration. Linear fits were used to extract second-order association rate constants (*k*_on_).

Equilibrium cyanide binding was strongly dependent on the oxidation state of hHb-based systems. Ferrous hHb showed no measurable cyanide binding within the tested concentration range (*K*_d,app_ > 50 μM), reflecting the low affinity of cyanide for ferrous heme. Consistent with this, ferrous Hb–Hp exhibited no detectable cyanide binding, confirming that ferric heme is required for effective ligand coordination.

Upon oxidation, cyanide affinity increased markedly. MethHb exhibited a *K*_d,app_ of 4.3 ± 0.6 μM,^41^ which is comparable to previously reported values for methHb (∼7.96 ± 1.53 μM),^30^ metHb-V (∼7.89 ± 0.36 μM),^30^ and oxidized tense quaternary state polymerized hHb (met-T-state PolyhHb, ∼3.9 ± 0.2 μM).^41^ The methHb–Hp complex showed a weaker apparent affinity (*K*_d,app_ = 21.8 ± 2.8 μM), indicating partial attenuation of equilibrium binding of cyanide within the complex, while still enabling measurable cyanide coordination.

Hill analysis indicated modest positive cooperativity for the oxidized species. MethHb displayed a Hill coefficient of 1.9 ± 0.2,^41^ consistent with literature values for methHb (∼2.42 ± 0.09),^30^ metHb-V (∼1.71 ± 0.07),^30^ and met-T-state PolyhHb (∼1.5 ± 0.1).^41^ The methHb–Hp complex exhibited a lower Hill coefficient (1.3 ± 0.1), suggesting slightly reduced cooperative behavior among heme sites in the bound methHb αβ dimers versus tetrameric methHb (α_2_β_2_).

Kinetic measurements were consistent with equilibrium trends. Ferrous constructs exhibited negligible cyanide association, while methHb displayed rapid binding (*k*_on_ = 82.7 ± 7.6 M^–1^ s^–1^),^41^ lower than reported values for methHb (∼187 M^–1^ s^–1^),^30^ metHb-V (∼173 M^–1^ s^–1^),^30^ and met-T-state PolyhHb (∼97.0 ± 12.2 M^–1^ s^–1^).^41^ The methHb–Hp complex exhibited a second-order rate constant of 134 ± 14 M^–1^ s^–1^, demonstrating fast ligand capture despite the complex’s larger size and decreased affinity.

Dissociation kinetics revealed strong retention of cyanide. MethHb exhibited a *k*_off_ of (3.5 ± 0.5) × 10^–4^ s^–1^,^41^ in line with reported values for mehtHb (∼0.984 × 10^–4^ s^–1^),^30^ metHb-V (∼2.02 × 10^–4^ s^–1^),^30^ and met-T-state PolyhHb (∼3.7 ± 0.4 × 10^–4^ s^–1^).^41^ The methHb–Hp complex showed dramatically slow dissociation kinetics (∼10^–3^ s^–1^), indicating highly stable cyanide sequestration once bound.

Overall, these results demonstrate that ferric heme availability is the key determinant of cyanide binding. MethHb, methHb–Hp, and literature-reported metHb, metHb-V, and met-T-state PolyhHb all exhibit strong binding when sufficient oxidation is achieved. The methHb–Hp complex is distinct in showing slightly reduced equilibrium affinity but faster association and highly suppressed dissociation, highlighting its potential for stable cyanide sequestration.

### 3.7 Azide Binding Kinetics

Azide binding kinetics were determined via equilibrium binding and rapid kinetic analysis via UV–visible spectroscopy and stopped-flow spectroscopy, respectively (shown in Figure 7).

**Figure 7:**
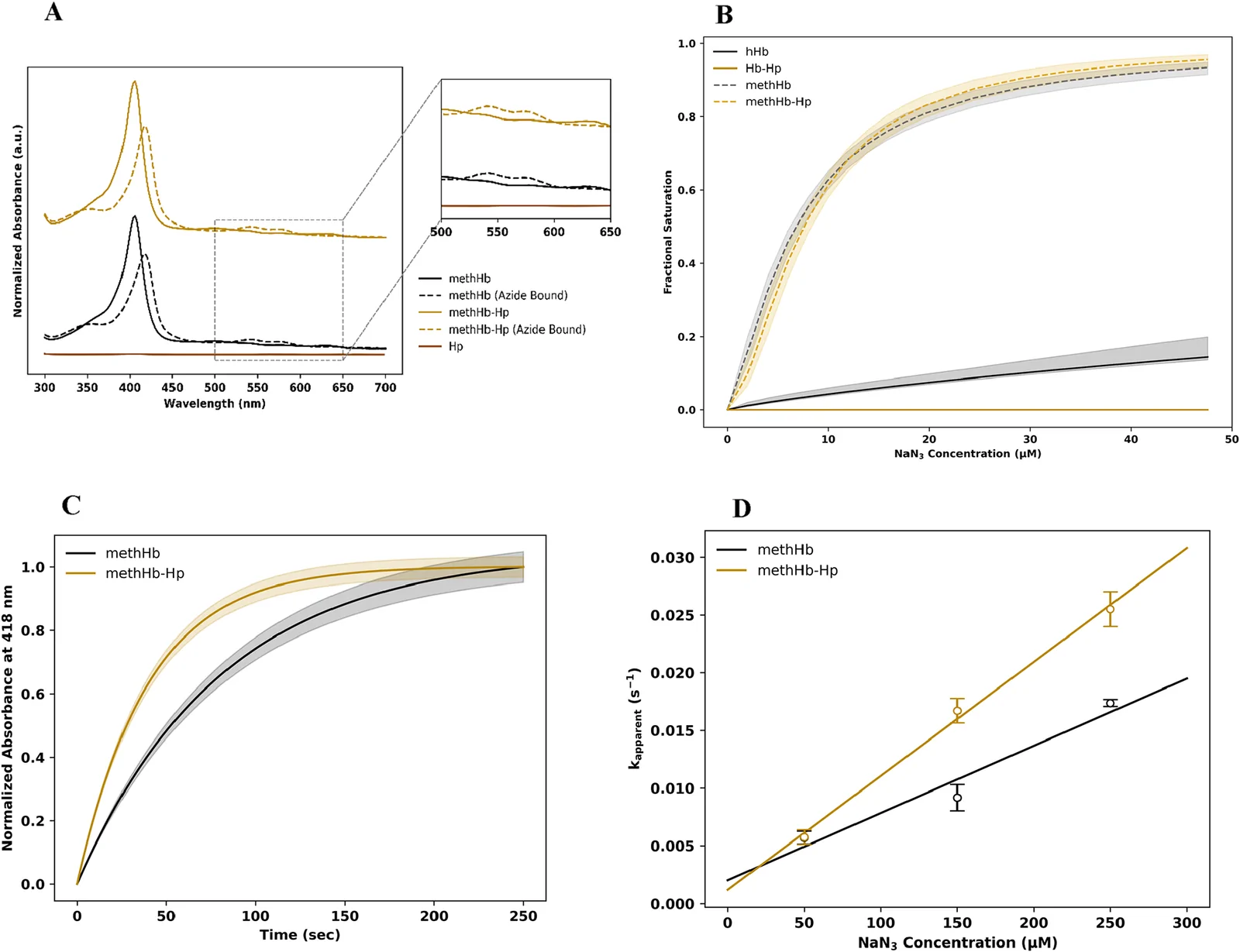
Equilibrium and kinetic analysis of azide binding to hHb-based constructs. (A) UV–visible spectra for methHb and methHb–Hp before and after azide binding. (B) Equilibrium azide binding curves for hHb, hHb–Hp, methHb, and methHb–Hp. Fractional saturation (*Y*) was calculated from changes in absorbance at 405 nm and plotted as a function of NaN_3_ concentration. Data were fit to the Hill equation to determine apparent dissociation constants (*K*_d,app_) and cooperativity coefficients where measurable. (C) Representative stopped-flow UV–visible spectroscopy kinetic traces for rapid binding of 100 μM azide with methHb and methHb–Hp. (D) Determination of azide association kinetics for methHb and methHb–Hp. Pseudo–first-order rate constants (*k*_app_), obtained from single-exponential fits of stopped-flow absorbance traces at 418 nm, were plotted against NaN_3_ concentration. Linear fits were used to extract second-order association rate constants (*k*_on_).

Equilibrium azide binding was strongly dependent on the oxidation state of hHb-based constructs. Ferrous hHb showed no measurable binding over the concentration range tested (*K*_d,app_ > 50 μM),^41^ consistent with the known low reactivity of ferrous heme toward azide. Similarly, ferrous hHb–Hp exhibited no detectable binding, confirming that ferric heme is required for effective ligand coordination.

Upon oxidation, azide affinity increased substantially. MethHb exhibited a *K*_d,app_ of 6.2 ± 0.6 μM,^41^ which is comparable to literature values for methHb (∼16.40 ± 1.77 μM),^30^ metHb-V (>25 μM),^30^ and met-T-state PolyhHb (∼7.9 ± 0.4 μM).^41^ The methHb–Hp complex displayed a similar affinity (*K*_d,app_ = 7.8 ± 0.3 μM), indicating that ligand access remains effective despite Hp binding.

Hill analysis indicated modest positive cooperativity for methHb (*n* = 1.4 ± 0.1),^41^ consistent with heterogeneous but functional ferric sites, and comparable to literature values for methHb (∼1.30 ± 0.09),^30^ metHb-V (∼1.09 ± 0.02),^30^ and met-T-state PolyhHb (*n* = 1.3 ± 0.1).^41^ The methHb–Hp complex exhibited a similar Hill coefficient (1.7 ± 0.1), suggesting similar cooperative behavior among heme sites.

Stopped-flow kinetic measurements supported the equilibrium trends. Ferrous constructs showed negligible association (*k*_on_ ≈ 0 M^–1^ s^–1^). MethHb exhibited rapid azide association (*k*_on_ = 58.3 ± 4.1 M^–1^ s^–1^),^41^ in line with literature values for methHb (∼83 M^–1^ s^–1^),^30^ metHb-V (∼61 M^–1^ s^–1^),^30^ and met-T-PolyhHb (36.7 ± 3.1 M^–1^ s^–1^).^41^ The methHb–Hp demonstrated a higher second order rate constant (98.8 ± 8.5 M^–1^ s^–1^) demonstrating faster ligand capture.

Dissociation kinetics indicated stable azide–heme complexes. MethHb exhibited a *k*_off_ of (3.6 ± 0.25) × 10^–4^ s^–1^, comparable with literature for mehtHb (∼5.22 × 10^–4^ s^–1^),^30^ metHb-V (∼6.82 × 10^–4^ s^–1^),^30^ and met-T-PolyhHb (3.6 ± 0.25) × 10^–4^ s^–1^.^41^ The methHb–Hp complex showed dramatically slow dissociation kinetics (∼10^–4^ s^–1^), indicating highly stable azide sequestration once bound.

Overall, these results demonstrate that ferric heme availability is the primary determinant of azide binding. MethHb, met-T-PolyhHb, and literature-reported metHb and metHb-V all exhibit strong binding when sufficient oxidation is achieved. The metHb–Hp complex is distinct in showing similar equilibrium affinity but faster association and highly stable ligand retention, highlighting its potential for effective azide sequestration.

### 3.8 Sulfide Binding Kinetics

Sulfide binding kinetics were determined via equilibrium binding and rapid kinetic analysis via UV–visible spectroscopy and stopped-flow spectroscopy, respectively (shown in Figure 8).

**Figure 8:**
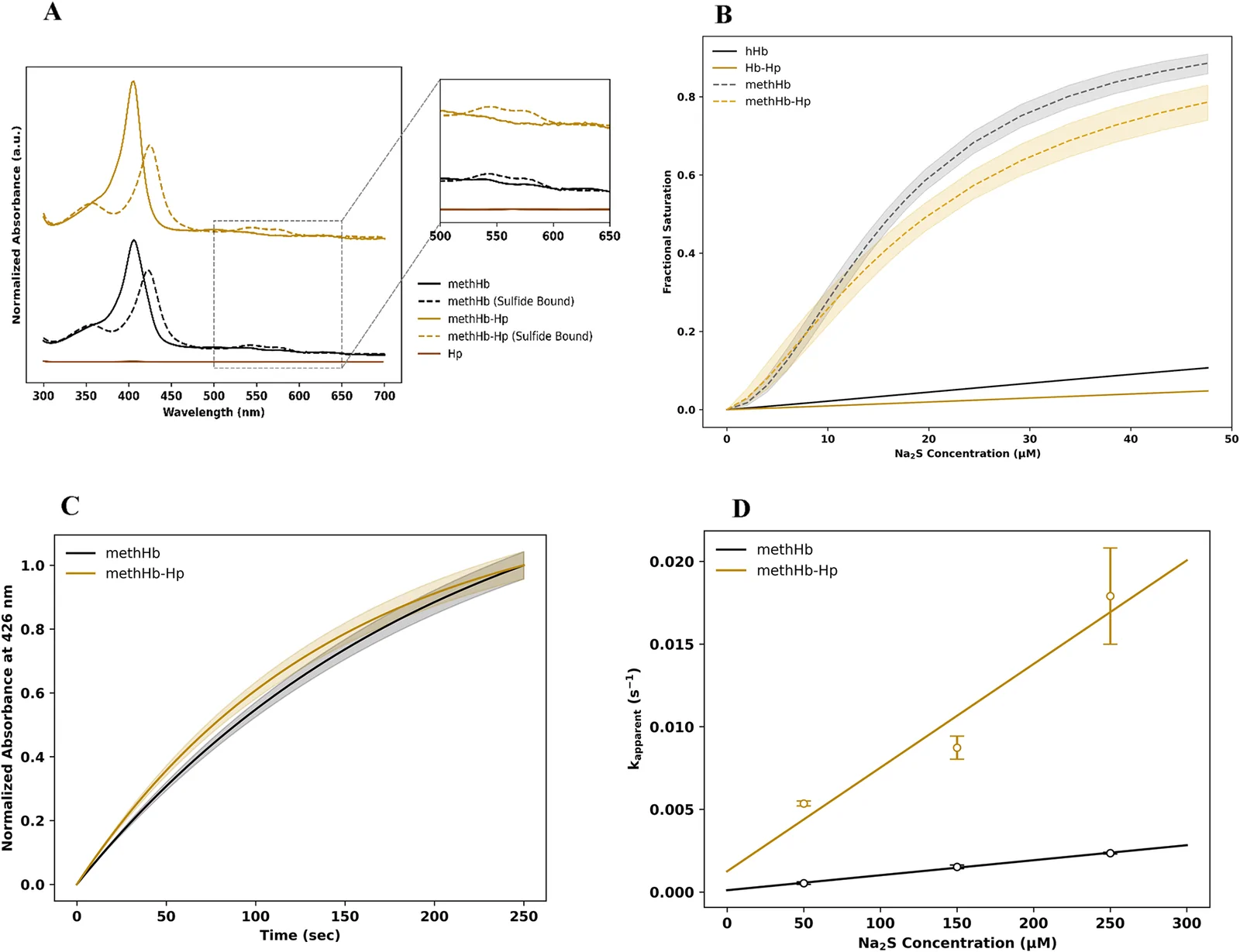
Equilibrium and kinetic analysis of sulfide binding to hHb-based constructs. (A) UV–visible spectra for methHb and methHb–Hp before and after sulfide binding. (B) Equilibrium cyanide binding curves for hHb, hHb–Hp, methHb, and methHb–Hp. Fractional saturation (*Y*) was calculated from changes in absorbance at 405 nm and plotted as a function of Na_2_S concentration. Data were fit to the Hill equation to determine apparent dissociation constants (*K*_d,app_) and cooperativity coefficients where measurable. (C) Representative stopped-flow UV–visible spectroscopy kinetic traces for rapid binding of 100 μM sulfide with methHb and methHb–Hp. (D) Determination of sulfide association kinetics for methHb and methHb–Hp. Pseudo–first-order rate constants (*k*_app_), obtained from single-exponential fits of stopped-flow absorbance traces at 426 nm, were plotted against Na_2_S concentration. Linear fits were used to extract second-order association rate constants (*k*_on_).

Equilibrium sulfide binding was strongly dependent on the oxidation state of hHb-based constructs and the availability of ferric heme. Ferrous hHb and ferrous Hb–Hp exhibited negligible binding over the concentration range tested (*K*_d,app_ > 50 μM), consistent with minimal sulfide affinity for ferrous heme.

Upon oxidation, sulfide binding increased significantly. MethHb exhibited a *K*_d,app_ of 16.0 ± 0.9 μM,^41^ which is slightly higher than literature values for methHb (∼5.09 ± 0.03 μM)^30^ and metHb-V (∼5.18 ± 0.94 μM),^30^ while met-T-PolyhHb showed a comparable *K*_d,app_ of 13.1 ± 2.6 μM.^41^ The methHb–Hp complex displayed a similar apparent affinity (*K*_d,app_ = 19.4 ± 1.2 μM), indicating that ferric heme sites remain accessible despite complexation.

Hill analysis revealed modest positive cooperativity for methHb (*n* = 1.9 ± 0.1)^41^ and met-T-PolyhHb (*n* = 1.1 ± 0.1),^41^ consistent with functional ferric binding sites, and comparable to literature values for methHb (∼1.30 ± 0.09)^30^ and metHb-V (∼1.09 ± 0.02).^30^ The methHb–Hp complex exhibited a similar Hill coefficient (1.5 ± 0.1), suggesting similar cooperative behavior among heme sites.

Stopped-flow kinetic measurements mirrored equilibrium trends. Ferrous constructs showed negligible association (*k*_on_ ≈ 0 M^–1^ s^–1^), while methHb exhibited rapid sulfide association (*k*_on_ = 9.4 ± 1.1 M^–1^ s^–1^).^41^ Met-T-PolyhHb displayed a similar rate constant (*k*_on_ = 9.2 ± 1.2 M^–1^ s^–1^), indicating that polymerization does not substantially impede ligand access.^41^ The metHb–Hp complex showed slightly slower kinetics (*k*_on_ = 63 ± 15 M^–1^ s^–1^), consistent with effective but partially sterically constrained binding. These values are lower than literature-reported methHb (∼867 M^–1^ s^–1^) and metHb-V (∼803 M^–1^ s^–1^), likely due to differences in sulfide donor ionization (Na_2_S vs NaHS), but they demonstrate that ferric heme drives effective ligand association.

Dissociation kinetics indicated stable sulfide–heme complexes for methHb and met-T-PolyhHb, with *k*_off_ values of (1.5 ± 0.17) × 10^–4^ s^–1^ and (1.2 ± 0.15) × 10^–4^ s^–1 41^, respectively, lower than literature values for methHb (∼14.28 × 10^–4^ s^–1^)^30^ and metHb-V (∼44.86 × 10^–4^ s^–1^),^30^ indicating tighter sulfide binding. The methHb-Hp complex exhibited remarkably slow dissociation kinetics of (1.5 ± 0.17) × 10^–3^ s^–1^ indicating highly stable sulfide sequestration once bound.

Overall, these results demonstrate that ferric heme availability is the dominant determinant of sulfide-binding performance. MethHb, met-T-PolyhHb, and literature-reported methHb and metHb-V all exhibit appreciable binding when sufficient oxidation is achieved. The metHb–Hp complex is distinct in showing similar equilibrium affinity but more rapid association and stable retention of sulfide, highlighting its potential for effective sulfide sequestration.

## 4 Conclusion

These findings establish that the ferric heme content in hHb is the primary determinant of effective cyanide, azide, and sulfide sequestration, and that Hp complexation to ferric hHb preserves–and may enhance–this capability while conferring substantial structure stabilization. The convergence of thermodynamic, kinetic, and structural data across three chemically distinct anions strengthens the mechanistic picture: the ferric heme in hHb is both necessary and sufficient for ligand capture, and Hp association to ferric hHb creates a more stable scaffold without disrupting the heme chemistry responsible for anion ligand capture. The methHb-Hp complex therefore emerges as a structurally resilient, broad-spectrum scavenger with favorable kinetic properties for neutralizing fast-acting mitochondrial toxins in therapeutic contexts.

The integration of equilibrium, kinetic, and structural measurements across four well-defined constructs and three ligands represents a methodological strength, allowing functional differences to be grounded in a consistent biophysical framework. However, these conclusions are drawn from *in vitro* measurements under idealized conditions, and the influence of competing plasma proteins, cellular redox environments, and *in vivo* pharmacokinetics on complex performance remains to be established in future preclinical work.
